# [Retracted] miR-378a-3p exerts tumor suppressive function on the tumorigenesis of esophageal squamous cell carcinoma by targeting Rab10

**DOI:** 10.3892/ijmm.2025.5640

**Published:** 2025-09-15

**Authors:** Naixin Ding, Xiujin Sun, Tingting Wang, Lei Huang, Jing Wen, Yiqin Zhou

Int J Mol Med 42: 381-391, 2018; DOI: 10.3892/ijmm.2018.3639

Following the publication of this paper, it was drawn to the Editor's attention by a concerned reader that certain of the flow cytometric data shown in Fig. 3 on p. 385 were strikingly similar to data which had been already been submitted for publication to several other journals that were written by different authors at different research institutes. In addition, overlapping data panels were noted with the EdU assay experiments in Fig. 2C, with the scratch-wound assay experiments in Fig. 10A, and with the cell invasion assay experiments in Fig. 10B, and the Rab10 western blot data in Fig. 9B for the EC109 cell line were strikingly similar to data that appeared in another journal by different authors at different research institutes.

Owing to the fact that the contentious flow cytometric and western blot data in the above article were found to be strikingly similar to data that had already been submitted for publication elsewhere, the Editor of *International Journal of Molecular Medicine* has decided that this paper should be retracted from the Journal. The authors were asked for an explanation to account for these concerns, but the Editorial Office did not receive a satisfactory reply. The Editor apologizes to the readership for any inconvenience caused.

